# Prospective virtual screening combined with bio-molecular simulation enabled identification of new inhibitors for the KRAS drug target

**DOI:** 10.1186/s13065-024-01152-z

**Published:** 2024-03-25

**Authors:** Amar Ajmal, Hind A Alkhatabi, Roaa M. Alreemi, Mubarak A. Alamri, Asaad Khalid, Ashraf N. Abdalla, Bader S. Alotaibi, Abdul Wadood

**Affiliations:** 1 Department of Biochemistry, Abdul Wali Khan University Mardan, Mardan, 23200 Pakistan; 2 Department of Biochemistry, College of Science, University of Jeddah, Jeddah, 21959, Saudi Arabia; 3 Department of Pharmaceutical Chemistry, College of Pharmacy, Prince Sattam Bin Abdulaziz University, Al-Kharj, 11942 Saudi Arabia; 4 Substance Abuse and Toxicology Research Center, Jazan University, P.O. Box: 114, Jazan, 45142 Saudi Arabia; 5 Department of Pharmacology and Toxicology, College of Pharmacy, Umm Al-Qura University, Makkah, 21955 Saudi Arabia; 6 Department of Clinical Laboratory Sciences, College of Applied Medical Sciences, Shaqra Univesity, Al- Quwayiyah, Riyadh, Saudi Arabia

**Keywords:** KRAS, Machine-learning, External validation, Molecular docking, Molecular dynamics simulation

## Abstract

Lung cancer is a disease with a high mortality rate and it is the number one cause of cancer death globally. Approximately 12–14% of non-small cell lung cancers are caused by mutations in KRAS^G12C^. The KRAS^G12C^ is one of the most prevalent mutants in lung cancer patients. KRAS was first considered undruggable. The sotorasib and adagrasib are the recently approved drugs that selectively target KRAS^G12C^, and offer new treatment approaches to enhance patient outcomes however drug resistance frequently arises. Drug development is a challenging, expensive, and time-consuming process. Recently, machine-learning-based virtual screening are used for the development of new drugs. In this study, we performed machine-learning-based virtual screening followed by molecular docking, all atoms molecular dynamics simulation, and binding energy calculations for the identifications of new inhibitors against the KRAS^G12C^ mutant. In this study, four machine learning models including, random forest, k-nearest neighbors, Gaussian naïve Bayes, and support vector machine were used. By using an external dataset and 5-fold cross-validation, the developed models were validated. Among all the models the performance of the random forest (RF) model was best on the train/test dataset and external dataset. The random forest model was further used for the virtual screening of the ZINC15 database, in-house database, Pakistani phytochemicals, and South African Natural Products database. A total of 100 ns MD simulation was performed for the four best docking score complexes as well as the standard compound in complex with KRAS^G12C^. Furthermore, the top four hits revealed greater stability and greater binding affinities for KRAS^G12C^ compared to the standard drug. These new hits have the potential to inhibit KRAS^G12C^ and may help to prevent KRAS-associated lung cancer. All the datasets used in this study can be freely available at (https://github.com/Amar-Ajmal/Datasets-for-KRAS).

**Supplementary Information:**

The online version contains supplementary material available at 10.1186/s13065-024-01152-z.

## Introduction

The most frequently mutated gene family in human malignancies is RAS including KRAS, NRAS, and HRAS. KRAS is one of the most prevalent isoforms among the RAS family, being found in 85% of pancreatic, 45% of colo-rectal, and 30% of lung cancer [1]. KRAS is a member of the broad family of proteins known as GTPases. KRAS is a plasma membrane-bound protein. KRAS functions as a molecular switch for downstream signal transduction by cycling between the on (GTP) and off (GDP) states [2]. Every year, about 50,000 people in the United States alone receive a new diagnosis of lung cancer caused by KRAS mutations [3]. Moreover, a history of smoking is frequently related to KRAS-driven lung malignancies [4]. The KRAS gene has six exons and is found on chromosome 12p11.1–12.2. KRAS is a small protein that is 21 KDa in size. The two domains of KRAS G and C domains, which are made up of six beta strands encircled by five alpha-helices, are the protein’s two functional domains. The C terminal domain is lipid-modified which helps in the membrane anchoring [5]. The G domain of KRAS is one of the functional biological areas which has 1-166 residues. Other crucial KRAS functional regions include the switch I and switch II domains, which operate as a binding interface for effector proteins. The Walker A motif, a short P-loop component with 10–14 residues, is present in the KRAS structure. In the P-loop or switch − 2 region the cancer mutation hotspots are found predominantly [6, 7]. The G-domain, a highly conserved region that contains switch I and switch II loops and is involved in GDP-GTP exchange, is one of the protein’s three primary domains [4]. KRAS transmits signals from the cell membrane to the nucleus when it is active, activating a variety of signaling pathways after receptor tyrosine kinase (RTK) activation (EGFR, ALK, or cMET) and ultimately causing the activation of transcription factors that regulate cell growth (cell proliferation and cell survival) and differentiation [8]. KRAS is activated when GTP binds to KRAS and causes alterations in the switch I and switch II loops of the G-domain. “KRAS-GTP directly interacts with and activates a number of downstream effector proteins in the active state, including RAF and PI3K”. A-RAF, B-RAF, and C-RAF are the three subtypes of serine-threonine kinase. The RAF in an active state activates MEK which activates ERK which promotes cell growth and proliferation [9]. The GTP and GDP-bound forms of the KRAS protein cycle during its 24-hour half-life with resynthesis [10]. KRAS was first considered undruggable due to lack of binding pocket which can be accessible to small molecules. However, sotorasib and adagrasib which just have recently been discovered and selectively target KRAS^G12^, offer new treatment approaches to enhance patient outcomes. Due to the development of sotorasib and adagrasib KRAS^G12C^ is now druggable [11]. Early adaptive feedback reactivation of signaling pathways has impeded prior attempts to target the RAS-RAF-MEK pathway and resulted in treatment resistance. Currently, in vitro and the clinical setting, have described that secondary KRAS mutations confer acquired resistance to KRAS^G12^ inhibitors [12]. Drug design and development is a challenging, expensive, and time-consuming procedure. It involves the discovery of promising targets and the design of therapeutically effective and safe drugs against promising targets. Computer-aided drug design (CADD), employs a number of computational and statistical techniques to efficiently assess biological target selection and hit identification [13, 14]. The process of drug development can be sped up by using advanced computational techniques. For the purpose of drug development, CADD can further make use of the integrated biochemical space to improve safety, and efficacy [15]. A number of machine learning algorithms are increasingly used in the drug development process. Only when reliable and accurate pre-processed data are combined with efficient computational methods and tools successful applications in the drug-designing process can be achieved [16] In this study, machine learning-based virtual screening was performed for the identification of new inhibitors against KRAS^G12^.

## Materials and methods

### Dataset preparation

The active compounds against KRAS^G12^ mutant with experimentally determined IC50 values were retrieved from the Binding DB [17]. The compounds in SDF format were imported to MOE software. Moreover, the DUD-E web database was accessed and the corresponding decoys were generated [18]. The class label was added to the dataset all the inactive compounds were labeled as 0 and the active compounds were labeled as 1. The entire dataset was split into train and test sets (70% and 30%) respectively [19]. Prior to train and test set splits 20% of the data was separated from the whole dataset which was used as an independent dataset for external validation.

### Molecular descriptors calculation and features selection

MOE software was used for 2D feature calculation [20, 21]. A total of 208 2D features were calculated. In order to avoid overfitting and to enhance the generalizability of the models, the dataset was preprocessed which included the removal of zero and not available (NA) values. To develop a model that is easy to understand and computationally cheap, it is essential to choose feature subsets that are most relevant to predictive targets. We used support SVM-RFE to choose features in order to collect as useful information as possible [22].

### Machine learning models

Four models including k-nearest neighbors, support vector machine, naïve Bayes, and random forest models were developed using open-source python v3.9. The Scikit-learn library of python was used for model development [23].

### K-nearest neighbor (kNN)

k-Nearest Neighbors (KNN) is a binary classification algorithm that classifies the data by calculating the distance between the nearest neighbors [24]. The number of neighbors considered for classification is represented by the parameter n_neighbors [25]. The best k value in this investigation was found as 11.

### Naïve Bayesian (NB)

The naive Bayesian is a reliable classification algorithm that is based on the Bayes theorem. A data set can be classified using the NB model under the suppositions that each attribute contributes equally and independently to a dataset [26]. In this work, python v3.9 was used for NB model generation.

### Random forest model

Breiman introduced the categorization algorithm known as RF [27]. Random Forest (RF) is a popular model that can be employed for data classification or regression tasks [28]. The tree of the random forest is trained using a bootstrap sample and the majority vote of the trees determines the predictions. The two primary hyperparameters that were optimized during model construction were max_features and n_estimators, which represent the number of trees constructed prior to predictions [25]. The number of estimations from 100 to 500 was taken into consideration.

### Support vector machine (SVM)

The SVM model is frequently used to solve the problems of classification, pattern recognition, and regression [29]. The multiclass classification problems can also be solved by SVM. SVM draws margin lines (support vectors) parallel to a separable hyper-plane between the data classes. To transform the low dimensional data to higher dimensional space SVM model use different kernel trick, which includes the linear, polynomial, sigmoid, and radial base function [30]. We used RBF and the grid search approach to determine the best values for the C and γ parameters. Finally, the optimal values were determined to be C = 1000 and γ = 1.

### Performance evaluation of models

Different performance evaluation parameters are used in machine learning to evaluate how effective an intelligent model is [31]. When a classification system generates true and false predictions, they are kept in a confusion matrix [32]. In many classification models, accuracy is used to evaluate the quality of the classification algorithm, but in some cases (such as with imbalanced datasets), accuracy alone is insufficient to assess a prediction model’s overall effectiveness [33]. The MCC parameter is considered an important indicator for measuring the performance of binary classification. The highest MCC value is an indication of the good performance of the model [34].

The receiver operating characteristic (ROC) curve is also effective to evaluate the performance of the models. A ROC curve can visually represent the true positive rate against the false positive rate [35]. In this study, various parameters such as sensitivity, specificity, accuracy, and MCC were calculated for the developed models. To further evaluate the performance of the best model the area under the ROC curve (AUC), which is used to rate the models, was also calculated. The perfect model has an AUC value of 1 while a value of 0.5 indicates the random performance of the model [36].

### Models validation

The three methods of validation most frequently employed by researchers are the independent tests, k-fold CV, and jackknife CV [37]. In order to evaluate the effectiveness of our models, we used five-fold cross-validation.

### Virtual screening and molecular docking study

The model with the best accuracy and MCC value was further used to screen a total of four databases including the Zinc database, the South African natural product database, Pakistani phytochemicals, and the in-house database. The hits predicted by ML algorithms were further docked against the KRAS^G12C^ mutant. For the molecular docking study, the PDB structure of the receptor KRAS^G12C^ mutant (PDB ID 6OIM) was retrieved from the RCS PDB database. As the target protein structure may be coupled to heavy atoms, water, ligands, and cofactors, it cannot be directly employed for molecular docking. Polar charges were added to the structure and the water molecules were removed [38]. The energy of the receptor was minimized using a gradient of 0.05. A total of 10 conformations were generated for each ligand. After docking completion, the best ligand conformations were evaluated for their binding interactions using PyMol software [39]. Furthermore, for covalent ligands, we employed the covalent docking protocol of MOE (2016) software. The Cys12 residue of the KRAS^G12C^ was defined as the reactive residue for covalent docking and Michael’s adduct reaction was used as the suitable reaction type for covalent docking [40].

### All atoms MD simulation

AMBER version 20 was used to perform the simulation of the top best docking score complexes. The ff14SB was used as the force field for the protein while the General Amber force field (GAFF) parameters were assigned to the ligands [41]. A TIP3P cubic box with an 8 Å distance around the protein complex was used for the MD simulations study. For the systems to be neutralized, counter ions like Na + or Cl ions were added. Energy minimization was done in two steps prior to MD simulations. In the first stage, 5000 steps of steepest descent were applied then conjugate gradient minimization was performed to gradually minimize the whole system. The system’s temperature was increased from 0 to 300 K during MD simulation with constant volume and periodic boundary conditions. All the systems were equilibrated for 3 ns with constant pressure and constant temperature [42]. Finally, a total of 100 ns MD simulation was performed for the top four protein-ligand complexes. Using the cpptraj module of AMBER 20 software, all of the generated MD trajectories were analyzed. The post-simulation analysis such as root mean square deviations (RMSD), root mean square fluctuations (RMSF), the radius of gyration (RoG), and dynamics cross-correlation map (DCCM) were performed using the CPPTRAJ module of Amber 20 after the completion of MD simulations [43].

### Binding energy calculation

The MMGBSA is the most significant approach in re-ranking the binding conformations [44]. In order to calculate the binding free energy of the KRAS^G12C^-ligand complexes by taking into account 2500 snapshots, we employed the MMPBSA.py script [45]. To estimate the binding free energy the following equation was used:

$$\Delta Gbind = \Delta Gcomplex - [\Delta Greceptor + \Delta Gligand]$$

∆Greceptor, ∆Gligand, and ∆Gcomplex represent the binding energies of proteins, drugs, and complexes, respectively while the ∆Gbind represents the total binding energy.

The individual binding energies that make up the overall binding free energy, such as those that are bonded (Gbond), electrostatic (Gele), polar (Gpol), and nonpolar (Gnpol), were estimated using the following equation.

$$G = {G_{bond}} + {G_{ele}} + {G_{vdW}} + {G_{pol}} + G$$

## Results

### Dataset preparation

A total of 386 active compounds for KRAS^G12C^ with reported IC50 values were retrieved from the binding databank database. The DUDE database [45] was accessed to generate the inactive compounds. A total of 1608 decoys were generated. By combining the active compounds and the decoys a dataset of 1994 compounds was prepared. The dataset was labeled with 1 and 0 indicating the active and inactive compounds respectively. From the whole dataset, 20% of the data was separated which was further used as an independent dataset for external validations of the ML models.

### Features calculation

MOE software was used to calculate a total of 208 2D descriptors. In order to avoid overfitting and enhance the generalizability of the model the dataset was preprocessed by removing zero and NA values present in the dataset. The number of features was reduced to 172 after preprocessing.

### Optimum features selection

**Fig. 1:**
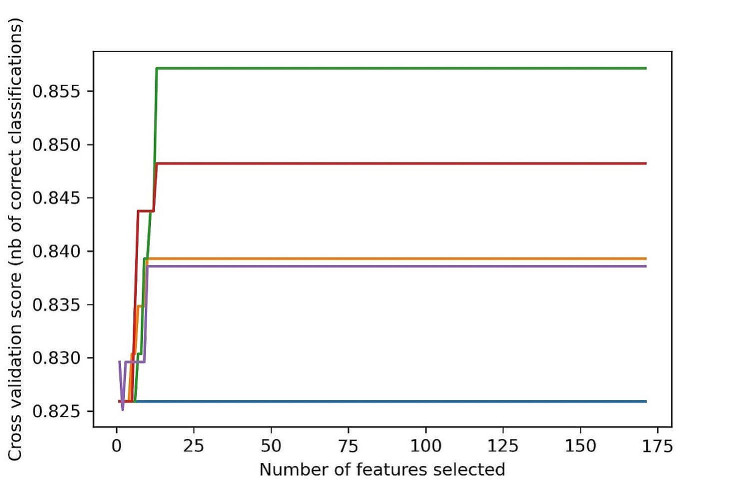
The feature selection curve for 2D molecular descriptors and the number of optimum features selected were 13

Filter, wrapper, and embedding approaches are the three types of methods currently used by the SVM to evaluate the significance of variables in the dataset. In the present study, we used recursive feature elimination (RFE), for the optimum features selection. The RFE is a gold standard method among wrapper techniques [46]. Out of 172 features, a total of 13 optimum features were selected. Figure 1 shows the optimum feature selection curve. The optimum features including PEOE_VSA + 2, PEOE_VSA_POS, PEOE_VSA + 0, PEOE_VSA-0, SlogP_VSA3, SMR_VSA6, vsa_hyd, PEOE_VSA_NEG, Weight, PEOE_VSA_HYD, Q_VSA_HYD, Q_VSA_POS, and vdw_area were selected using the SVM RFE technique. To improve each model’s performance, selected subsets of features were used to train all machine learning models.

### Chemical space and diversity

**Fig. 2:**
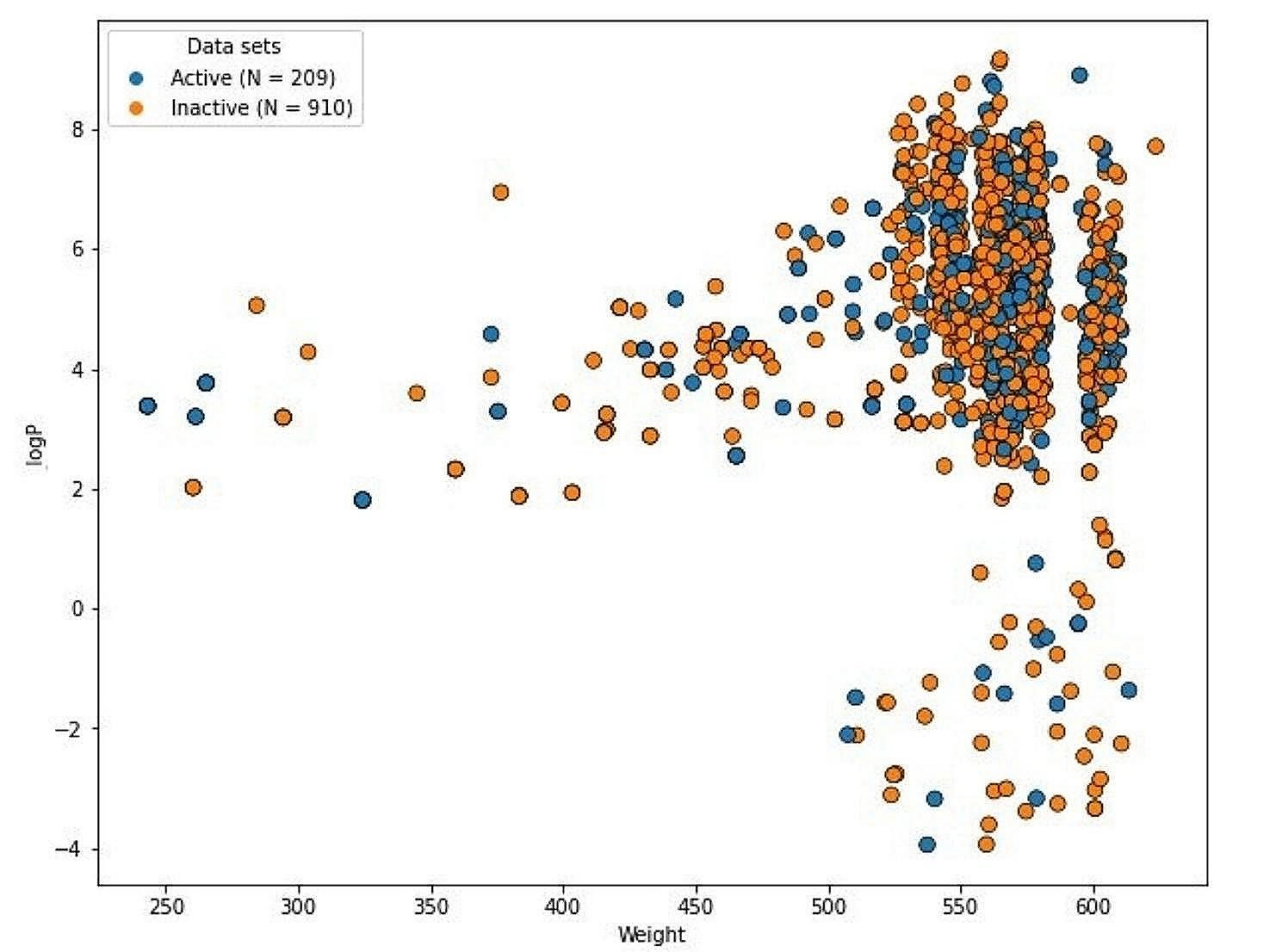
Chemical diversity distribution of the training set. The X-axis defined the molecular weight and Y-axis shows logP

**Fig. 3:**
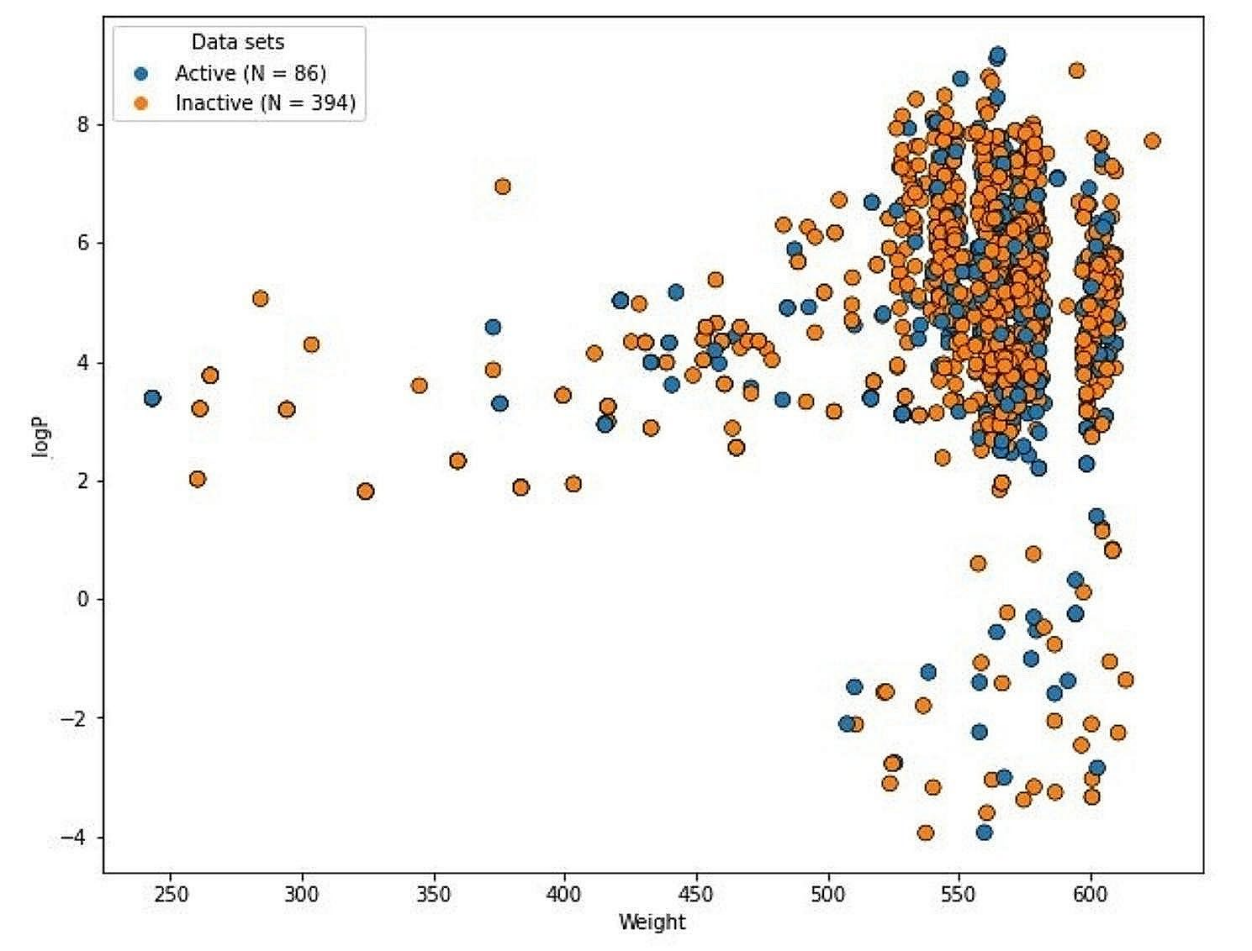
Chemical diversity distribution of the test set. The X-axis defined the molecular weight and Y-axis shows logP

The reliability of ML algorithm depends on the chemical diversity of a dataset. To execute the models perfectly, substantial chemical space is required. Figures 2 and 3 displays the significant chemical space between logP and molecular weight (MW) for the train and test set respectively. A significant chemical gap between inhibitors that are active and those that are not, with logP and MW varying from 4 to 8 and 250–600 Da was found for both the train and test datasets.

### Performance of machine learning models

**Fig. 4:**
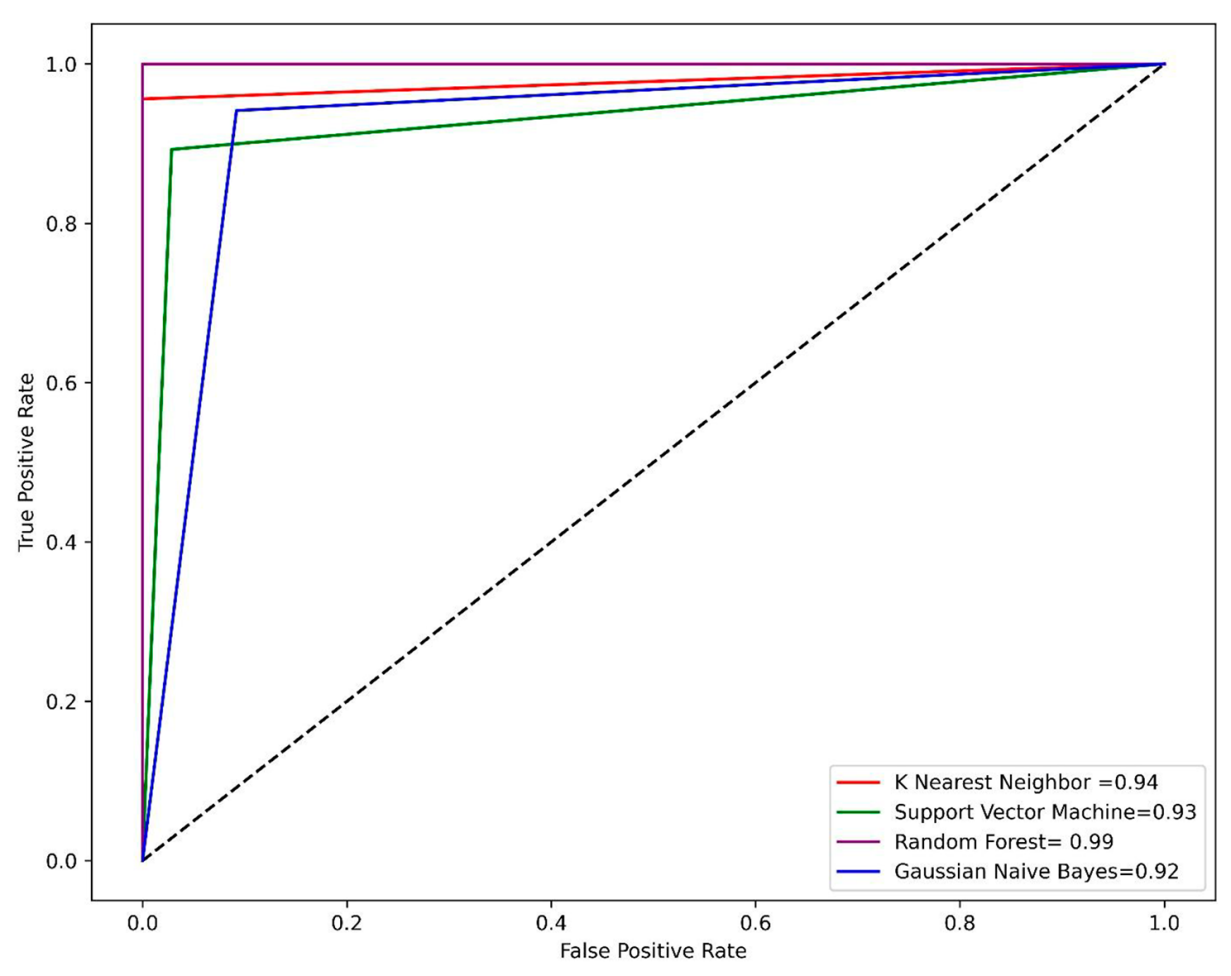
The AUC-ROC curve on the train set for all four models

**Fig. 5:**
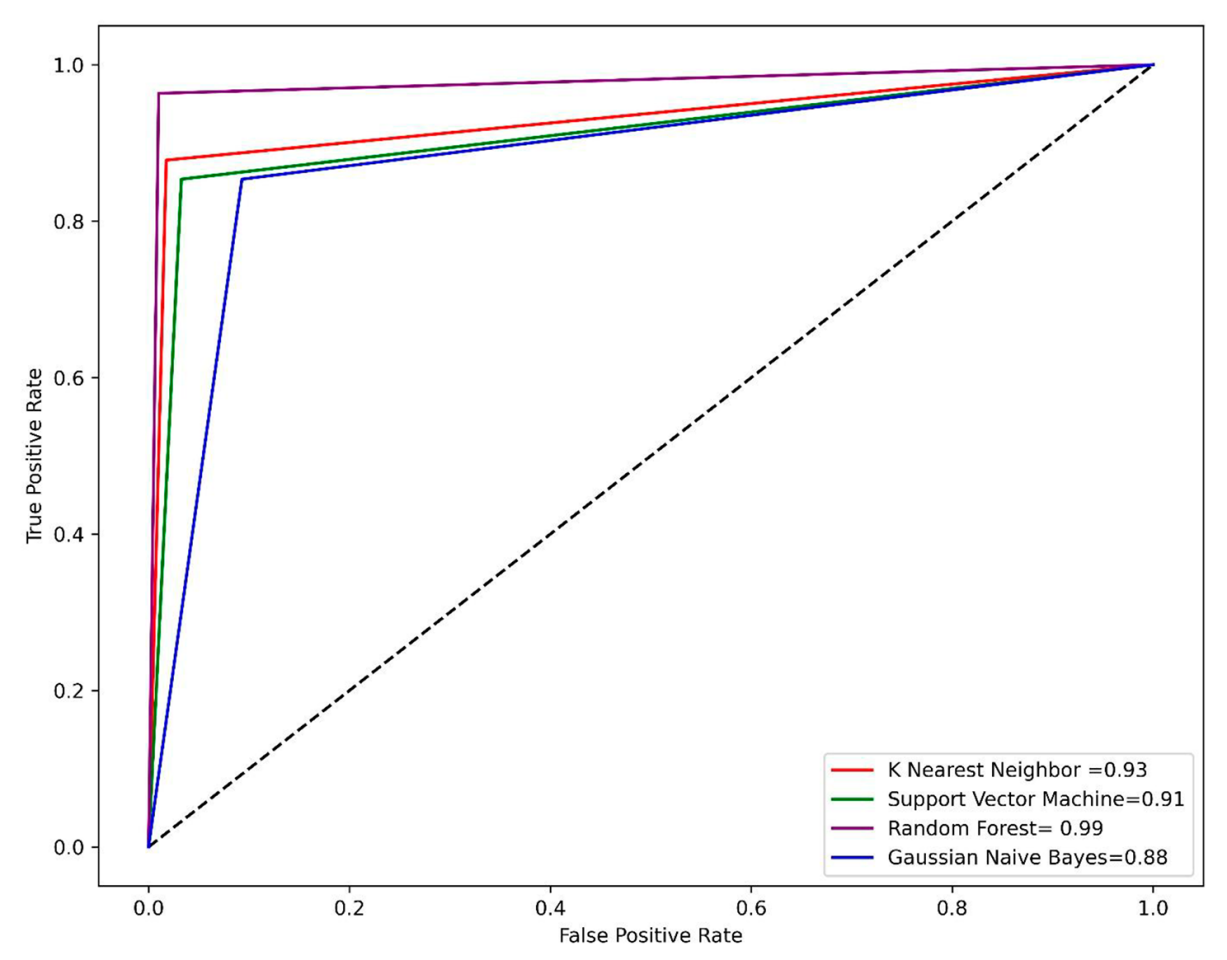
The AUC-ROC curve on the test set for all four models

The dataset was split into train and test sets with 70% of the data considered as a train set while 30% of the data was selected as a test set. Open-source python v3.9 was used for model generation. A number of supervised machine learning models including KNN, SVM, GNB, and RF were applied. To evaluate the model performance different parameters such as accuracy, sensitivity, specificity, and MCC were calculated. The RF model was found as the best model its performance was best on both the train and test set. The accuracy of RF model was 98% on both the train and test sets. The MCC value of the RF model was 0.95 on the train set and 0.97 on the test set. The accuracy of the KNN and SVM models was 94% on both the train and test sets while the MCC of the KNN model was 0.82 on the train set and 0.87 on the test set. The accuracy of the GNB model was 92% on the train set and 89% on the test set. The overall performance of the four generated models on the train set is summarized in Table 1 while Table 2 describes the performance of the four models on the test set. One of the most reliable methods for evaluating the model performance is the analysis of the ROC-AUC curve. The RF model has achieved the highest area under the curve (AUC) value of 0.99 on both the train and test set, followed by KNN with an AUC value of 0.94 on the train and 0.93 on the test set. Figure 4 represents the ROC-AUC curve on the train set while Fig. 5 represents the ROC-AUC curve on the test set.

**Table 1:** Performance of ML models on the train set

Model	Accuracy	Sensitivity	Specificity	MCC	Precision	Recall
KNN	94%	0.95	0.94	0.82	0.74	0.86
SVM	94%	0.92	0.95	0.84	0.83	0.90
RF	98%	0.96	0.99	0.95	0.97	0.97
GNB	92%	0.85	0.94	0.77	0.70	0.90

**Table 2:** Performance evaluation of ML models on the test dataset

ML model	Accuracy	Sensitivity	Specificity	MCC	Precision	Recall
KNN	96%	0.87	0.98	0.87	0.86	0.89
SVM	94%	0.85	0.96	0.81	0.81	0.86
RF	98%	0.95	0.99	0.97	0.93	0.95
GNB	89%	0.85	0.90	0.68	0.80	0.93

### Models validation by independent dataset

A classification model’s predictive power is not only assessed by its MCC score and accuracy during internal validation. As a result, any machine learning-based model’s performance is dependent both on internal and external validation. For external validation of the models, an independent dataset was prepared. A total of 397 compounds were added to the dataset with a total of 99 compounds as active against KRAS^G12C^ while the remaining were inactive compounds in the independent dataset. The inhibitors of the external dataset were not present in the train or test sets. As compared, other ML algorithms the RF model revealed the highest accuracy, sensitivity, specificity, and MCC values on the independent dataset (Table S1, Table S2). Since the RF model performs the best among all of the models, so it was used for virtual screening to find potent KRAS^G12C^ inhibitors. The ROC-AUC curve for the independent dataset is presented in Figures S1 and S2.

### Virtual screening

Four lakh compounds retrieved from the ZINC15 database were passed from the Lipinski rule of 5 before the virtual screening. Among the 4 lakh compounds, only 1 lakh compounds were found to obey the Rule of five. Only sixty thousand were non-toxic so only these compounds were selected for the virtual screening. The updated version of the South African natural product database contained a total of 1012 compounds that were used for virtual screening. Furthermore, a total of five thousand Pakistani phytochemicals retrieved from the PubChem database and a total of 2 thousand compounds from the in-house database were used for the virtual screening. A total of 101 compounds from the ZINC15 database, 42 compounds from the Pakistani phytochemicals, 23 compounds from the in-house database, and 19 compounds from the SANCDB (South African Natural Compounds Data Base) were predicted as active by the RF (Random Forest) model.

### Molecular docking analysis

The compounds predicted as active by the RF model were docked against the KRAS^G12C^ mutant. The docking study revealed that among the 101 active compounds of the ZINC database, the docking score of most of the compounds was good. The compound ZINC001458505494 was predicted as the covalent inhibitor of KRAS G12C. The docking score of compound ZINC001458505494 was found as -7.80. The compound ZINC001436082395 established a total of four conventional hydrogen bond interactions with Pro34, Tyr32, Lys16, and Ala59 residue of the receptor while one covalent bond with Cys12 and one Pi-H contact with Gln61 was also observed. Compound ZINC001436082395 with an S score of -12.15 formed one five H-bond interactions with Ser65, Glu62, Gly60, Gln61, and Ala66. Table S3 describes the interaction pattern of the top four best compounds along with their docking score while Table S4 describes the drug-like properties of the top five best docking-scored compounds of the ZINC database.

Twenty-three hits were found as active against KRAS^G12C^ from the virtual screening of the in-house database. The docking analysis of these 23 compounds revealed that H-209 is the most potent with an S score of -16.16. The compound H-209 formed four H-bond interactions with the Cys12, Asn86, Lys88 residues, and one Pi-H contact with the Cys12 residue of KRAS^G12C^. The compound H-164 with a docking score of -14.14 was found as the second potent. The compound H-164 formed hydrogen bond interactions with Gln99, Glu91, Glu62, Arg102, and Lys88. Compound H-237 formed a similar pattern of interaction with Arg102, Glu98, and Glu91 as established by the compound H-164. However, compound H-237 formed two pi-pi interactions with His95 cryptic pocket residue and His94. The interaction pattern of the top five best docking score compounds is present in Table S5 while the drug-like properties of the top 5 best compounds of the in-house database are present in Table S6.

The virtual screening of the South African Natural Products database revealed a total of 19 compounds out of 1012 compounds as active against the KRAS^G12C^. Among the 19 docked compounds the compound SANC00905 was the most promising covalent inhibitor with an S score of -9.61. The most potent compound SANC00905 revealed a total of five hydrogen bond interactions with Cys12, Glu62, Gln99, and Arg68 residues of the KRAS^G12C^. It was also found that the SANC00905 formed one covalent bond with the mutated Cys12 residue of KRAS G12C and one H-pi contact with the His95 cryptic pocket residue of the KRAS G12C protein. The interaction pattern of the top six best docking-scored compounds is present in Table S7 while the properties of the best docking-scored compounds of the SANCDB are present in Table S8. Furthermore, 42 compounds were identified as active out of the total five thousand Pakistani phytochemicals. Among the docked compounds the compound PubChem ID 11,968,893 was predicted as the most promising with a docking score of -18.58. The potent compound PubChem ID 11,968,893 formed a total of seven hydrogen bond interactions with Cys12, Gln61, Glu62, Asn86, and Lys88 residues while one Pi-H contact was also observed with the Lys88. The interactions and docking scores of the most promising Pakistani phytochemicals are present in Table S9 and their drug-like properties are present in Table S10. Overall our molecular docking study revealed two covalent inhibitors (ZINC001458505494 and SANC00905) and two non-covalent inhibitors (H-209, and PubChem ID 11,968,893) for the KRAS G12C drug target. The 3D interactions of the covalent inhibitors in complex with KRAS^G12C^ are present in Fig. 6 (A) while 3D interactions of the non-covalent inhibitors are shown in Fig. 6 (B). The 2D interactions of the best docking score compounds from all databases are shown in Figure S3. Table S11 displays the docking result of the covalent inhibitors.

**Fig. 6A:**
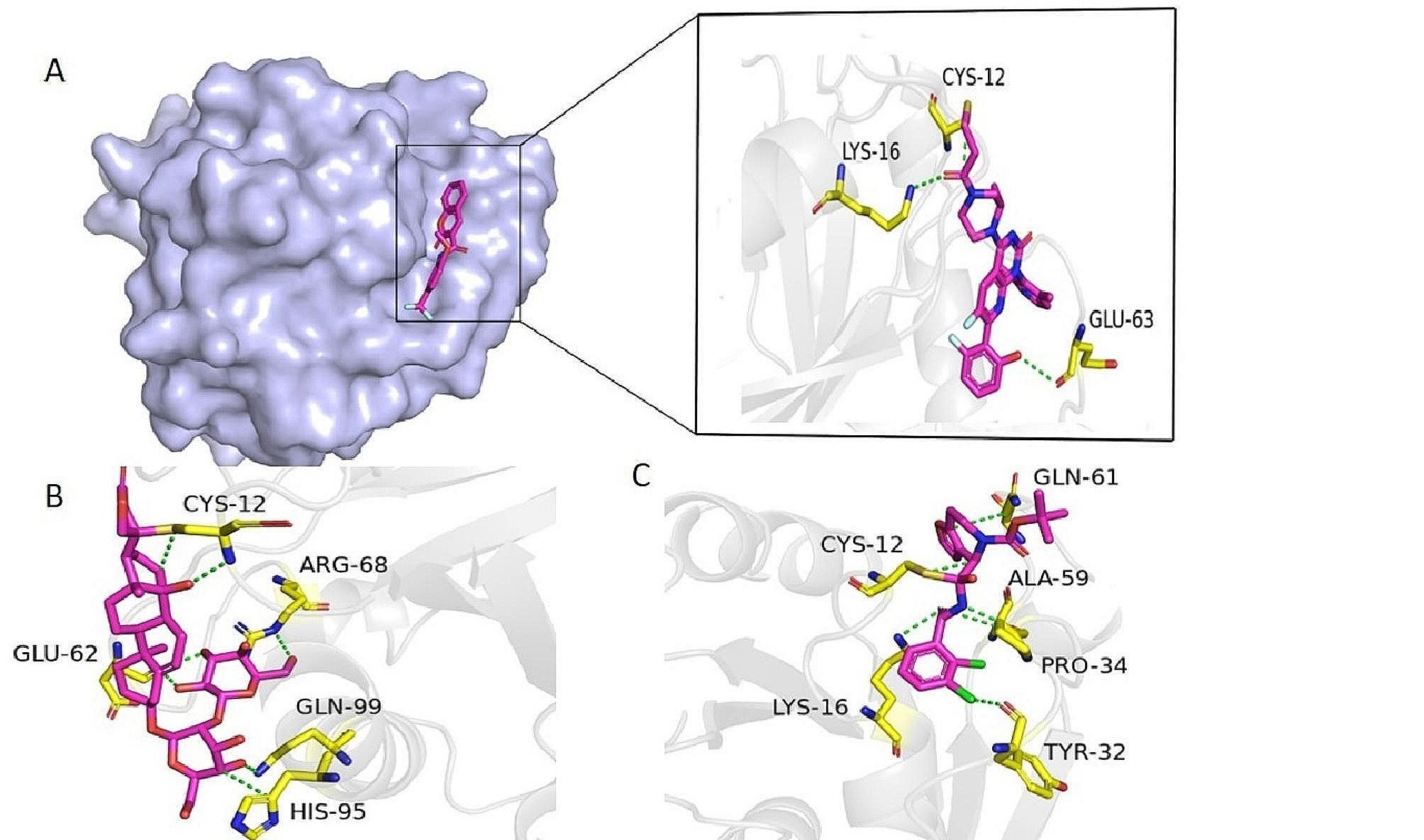
3D interactions of covalent inhibitors (**A**) standard drug- KRAS^G12C^ complex (**B**) SANC00905- KRAS^G12C^ (**C**) ZINC001458505494- KRAS^G12C^

**Fig. 6B:**
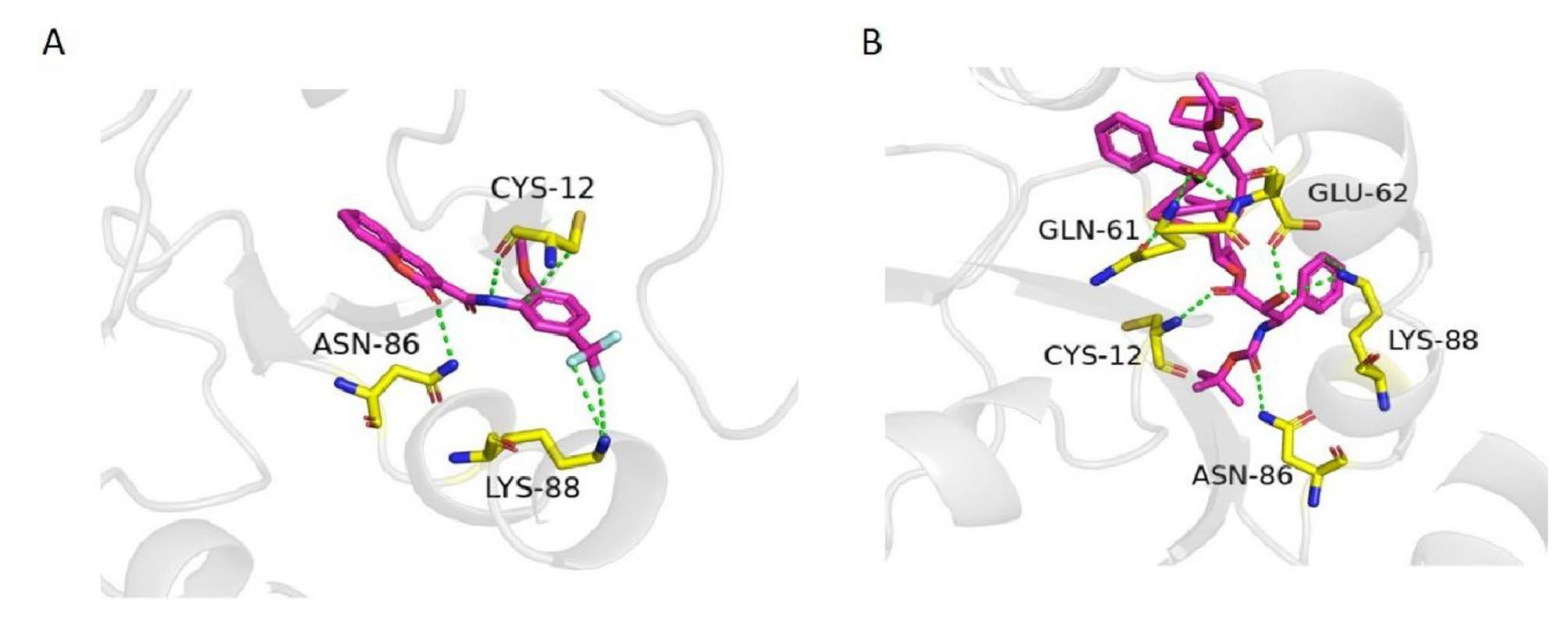
3D interactions of non-covalent inhibitors (**A**) H-209- KRAS^G12C^ (**B**) PubChem-CID11968893-KRAS^G12C^

### Post simulation analysis

#### RMSD analysis

The stability of KRAS^G12^ in complexes with the top four ligands was evaluated from the RMSD analysis during the 100ns MD simulation. The stability of complexes and details regarding the structural conformation during the simulation can be provided by the RMSD value. Figure S4 represent the RMSD plots for the top four best docking-score protein-ligand complexes and the standard drug sotorasib in the complex with the KRAS^G12C^. All four complexes were stable during the MD simulation. In Replica-1 no significant deviations were observed in the complex H-209- KRAS^G12C^ during the simulation. Some minor fluctuations were observed during 5–15 ns and 20–35 ns but after that, the complex remained stable till the 100 ns simulation (Figure. S4). In Replica-2 only minor deviations were seen during 20–25 ns in the H-209 complex after that the RMSD converged and a stable behavior was observed during the entire 100 ns MD simulation. The RMSD of complex PubChem-CID11968893-KRAS^G12C^ was highly stable in Replica-1, and only minor fluctuations during the 25–30 ns and 45–50 ns were observed after that the complex reached stability and remained stable till 100 ns. A similar pattern of RMSD was observed for PubChem-CID11968893-KRAS^G12C^ in Replica-2. The system was found to be highly stable in both runs (Figure. S4). The complex SANC00905- KRAS^G12C^ revealed stable behavior during the simulation. However, some minor deviations during the 23–38 ns were reported in Replica-1, and after that the system gained stability and remained stable during the 100 ns MD simulation. In Replica-2 the complex SANC00905- KRAS^G12C^ revealed a more stable behavior and only minor deviations were seen during 25–38 ns after that no major or minor deviations were observed till 100 ns. Initially, the RMSD of complex ZINC001458505494- KRAS^G12C^ was high during the first 10 ns afterward the RMSD decreased and reached stability but only minor deviations during 42–45 ns and 94-98ns were found in Replica-1 and the overall RMSD was stable. In Replica-2 the RMSD of complex ZINC001458505494- KRAS^G12C^ was highly stable except 80–90 ns. The RMSD of the standard drug in complex with KRAS^G12C^ was initially stable in Replica-1 but then some major fluctuations were found during the 60-65ns and 70–85 ns after that the system attain stability till 100ns in both the Replica.

#### RMSF analysis

Flexibility at the residue level can be analyzed from the RMSF profile. The flexible and stable regions are represented by a higher and lower RMSF value respectively [47]. The complexes almost showed the same pattern of residual flexibility in Replica-1 and Replica-2. The residues 1–29, 69–100, and 105–170 revealed high stability during the MD run. A total of three different peaks were found at three different time periods (Figure S5). Initial peak was observed with ASP30, GLU31, TYR32, PRO34 and THR 35, second with GLU62, GLU63, TYR64, SER65, ALA66, MET67, and ARG68 and third with ARG102, VAL103 and LYS104 residues. The residues that revealed great fluctuations were not the active site residues except Glu63. On the other hand, the residues lying in the active site were found as highly stable. Figure S5 display the RMSF pattern of Replica 1 and Replica 2 for all the systems.

#### Compactness analysis

To comprehend the degree of compactness of each ligand-bound system the Gyration radius (RoG) was determined. To evaluate how these ligands remained intact with the KRAS^G12C^ during the 100 ns MD simulation the RoG was calculated. Moreover, the compactness of systems represents stability. A lower RoG value denotes greater stability and a high RoG value denotes an unstable system [48]. In Replica-1 and Replica-2 the average RoG value was determined as 15.6–15.9 Å for the H-209-KRAS^G12C^ complex. The average RoG of the PubChem-CID11968893-KRAS^G12C^ system was 15.3–15.8 Å in Replica-1 while in Replica-2 the RoG was found to be 15.2–15.5 Å and the complex was observed as highly compact with no major deviations. The average RoG of the SANC00905-KRAS^G12C^ complex was 15.2–15.6 Å in Replica-1 while in Replica-2 a similar pattern of the RoG was found. The RoG of the ZINC001458505494-KRAS^G12C^ was found as 15.6–16.6 Å in Replica-1 while in Replica-2 the average RoG was found to be 15.4–16.4 Å. The RoG of the standard drug- KRAS^G12C^ complex was initially compact but increased from 15.2 to 16.4 Å during the 80-90ns then decreased after 90ns and remained compact till 100 ns. As compared to all other complexes the standard drug in complex with KRAS^G12C^ revealed a little unstable behavior. Among all the complexes the compound SANC00905 found as the covalent inhibitors for KRAS G12C was more compact during MD simulation. Figure S6 displays the RoG plots of all the ligand-bound complexes.

### Dynamic cross-correlation map (DCCM)

**Fig. 7:**
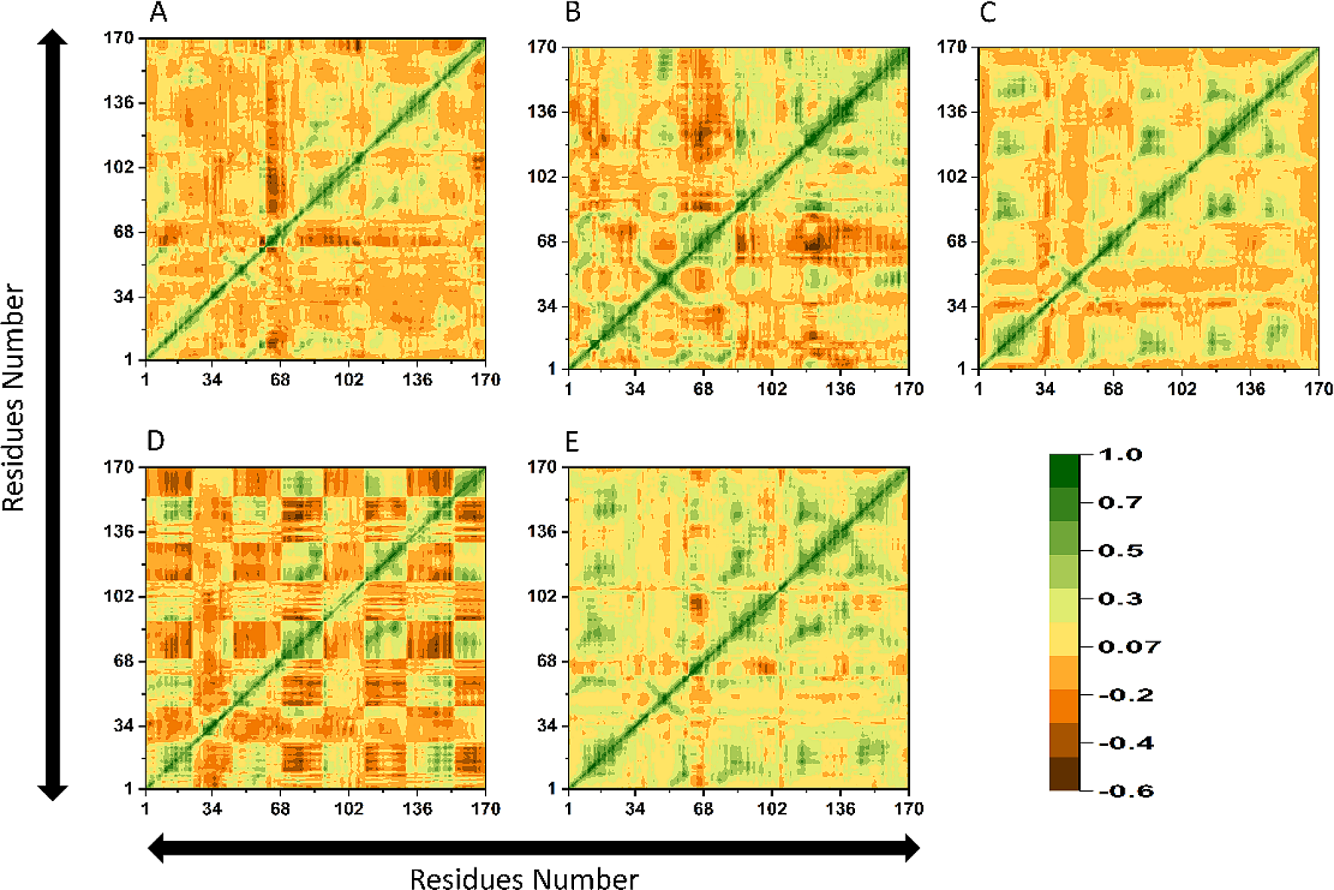
The DCCM map of (**A**) H-209- KRAS^G12C^ (**B**) PubChem-CID11968893-KRAS^G12C^ (**C**) SANC00905-KRAS^G12C^ (**D**) ZINC001458505494-KRAS^G12C^ (**E**) standard drug-KRAS^G12C^ complex. The X-axis and Y-axis display number of residues

The negative correlations imply that residues move in the opposite direction, and positive correlations show that residues are moving in the same direction i.e. anti-parallel and parallel direction. The residues displayed a positive correlation suggesting that the positive correlation may be caused by ligands interactions with the active site residues of KRAS. The green color indicates positive correlation while the dark brown color revealed negative correlation among the residues (Fig. 7). Among all the simulated systems PubChem-CID11968893-KRAS^G12C^ complex and SANC00905-KRAS^G12C^ have the highest positive correlation motions followed by the complex ZINC001458505494-KRAS^G12C^. Additionally, the H-209-KRAS^G12C^ complex exhibits greater correlated and anti-correlated motion as compared to the control.

### Binding energy calculation

It was found that all of the predicted active ligands in the complex with KRAS^G12C^ displayed strong binding affinity as compared to the control system, indicating that all of the systems are more likely to be stable. Table 3 provides an overview of the binding free energy and its components determined by the MM-GBSA calculation. According to the findings, the SANC00905-KRAS^G12C^ complex had the highest negative binding free energy value (-53 kJ/mol), followed by the PubChem-CID11968893-KRAS^G12C^ complex (-50 kJ/mol). As demonstrated in Table 3, all four predicted ligand complexes had very good binding free energy values, compared to the control indicating that they were more stable during the simulation. The total binding energy for sotorasib- KRAS^G12C^ on the other hand, was predicted to be around − 45 kcal/mol. These findings clearly imply that these ligands have more potent inhibitory potential than the standard drug.

**Table 3:** MMGBSA analysis indicating binding energy of all the complexes

Complex (Replica-1)	vdW	EEL	ESURF	EGB	ΔG TOTAL
H-209-KRAS^G12C^	-55.26	-9.38	-4.51	28.34	-45.82
ZINC001458505494-KRAS^G12C^	-61.41	-11.74	-6.01	26.56	-47.61
SANC00905-KRAS^G12C^	-71.52	-20.78	-9.10	18.70	-53.71
PubChem-CID11968893-KRAS^G12C^	-69.60	-17.03	-8.53	23.89	-50.28
Sotorasib-KRAS^G12C^	-52.47	-7.00	-4.06	29.21	-45.42
**Complex (Replica-2)**	**vdW**	**EEL**	**ESURF**	**EGB**	**ΔG TOTAL**
H-209-KRAS^G12C^	-48.78	-9.82	-5.86	25.38	-39.08
ZINC001458505494-KRAS^G12C^	-42.78	-5.46	-4.81	17.82	-35.24
SANC00905-KRAS^G12C^	-66.44	-4.81	-6.86	20.80	-57.33
PubChem-CID11968893-KRAS^G12C^	-66.58	-10.66	-6.66	32.41	-51.50
Sotorasib-KRAS^G12C^	-51.98	-11.82	-6.09	29.10	-40.80

vdW = van der Waals energy, EEL = electrostatic energy, ESURF = surface areas energy, EGB = the electrostatic contribution to the solvation free energy

## Discussion

Approximately 1.8 million people die each year due to lung cancer and lung cancer is considered the number one cause of cancer death worldwide with a high mortality rate. Approximately 84% of all lung malignancies are non-small cell lung cancers (NSCLC). The five-year survival rate of NSCL cancer patients is only 25% [49]. The KRAS^G12C^ gene mutation is a key initiator of NSCLC [50]. The clinical response rates of the developed drugs sotorasib and adagrasib are high, and toxicity from these drugs is low. However, resistance often develops after a few months of treatment [50]. Researchers are now searching for new effective drugs. The process of finding new drugs has substantially advanced through the use of ML algorithms. The use of multiple ML algorithms in drug discovery has considerably benefited pharmaceutical industries. These algorithms are frequently used in predicting the bioactivity of molecules, predicting drug-protein interactions, and optimizing the bioactivity and safety profile of the molecules [51]. A number of studies have been conducted on ML-based virtual screening [52, 53]. For instance, in our previous studies, we carried out ML-based virtual screening for the identification of new inhibitors against STAT3 a cancer drug target, and the Main protease drug target in the SARS CoV-2 [20, 54]. Similarly, some researchers used ML-based models for the identification of the functional groups responsible for binding [55]. A previous study used various ML algorithms for the identification of the new inhibitors from the mangrove secondary metabolic natural products database against KRAS^G12C^ protein [56].

In this study, we also used ML-based virtual screening to predict new inhibitors against KRAS^G12C^. Different machine-learning models including the ensemble RF model, KNN, SVM, and GNB were used for the classification purpose. The performance of all the proposed algorithms was evaluated by the parameters such as accuracy, sensitivity, specificity, and MCC. The performance of all the models revealed that the ensemble RF model was the best by achieving an accuracy of 98%. The RF model was further used for the virtual screening of in-house, ZINC, Pakistani phytochemicals, and South African Natural Products databases. Phytochemicals, or naturally occurring plant molecules, are important sources of new drug discovery and are also used to treat cancer. These phytochemicals frequently work by controlling molecular pathways that are connected to the development and spread of cancer. The precise processes include boosting antioxidant status, inhibiting carcinogens, reducing proliferation, and inducing cell cycle arrest and apoptosis [58]. The previous study reported that the phytochemicals are effective against a variety of diseases including, diabetes, TB, skin infections, malaria, anemia, and epilepsy [57]. The hits predicted by the RF model were further docked against the KRAS^G12C^. The docking results revealed a number of compounds with good docking scores and interactions with KRAS^G12C^ as compared to the standard drug sotorasib. Most of our predicted compounds revealed a similar pattern of interactions to the previous molecular docking studies carried out for KRAS^G12C^. In the previous study two most promising compounds such as compound 14 and compound 44 revealed interactions with Cys12, Lys16, Pro34, Gly60, and Arg68 residues of the receptor [56]. Our compounds also established interactions with Cys12, Lys16, Pro34 and Gly60. Similarly, in another study compound CID_146235508 was found the most potent compound against KRAS^G12C^. This compound made hydrogen bond and hydrophobic contacts with Cys12, Glu63, Lys16, Met72, Arg68, Ala59, and Tyr96 residues of the receptor [39] and a similar pattern of interactions was found in our study. After molecular docking, 100 ns MD simulation was performed for the top four complexes to reveal dynamic changes and the stability of the complexes. The RMSD analysis indicated the stable binding of the predicted compounds with the protein indicating these compounds as suitable inhibitors against the KRAS^G12C^. The RoG analysis which are in line with the RMSD profile, further supported the complex SANC00905- KRAS^G12C^ stability compared to all complexes. The calculated binding free energy for the four complexes and the control revealed that the binding energy of all the complexes was lower as compared to the standard drug which clearly indicates that these compounds can bind strongly with the receptor and can make more stable complexes with the KRAS^G12C^.

## Conclusion

Targeting KRAS^G12C^ is found to be viable in anti-cancer research. In this study, both synthetic and natural compounds were screened against KRAS^G12C^ using machine-learning algorithms. To find the new hit compounds with the strongest anti-cancer potential, four databases such as ZINC, in-house, Pakistani phytochemicals, and South African Natural Products databases were screened. Using the molecular docking study the hits predicted from virtual screening were further analyzed for interactions with KRAS^G12C^. Based on the interaction, the compounds that revealed good binding interactions were selected for MD simulation and binding energy calculation. As compared to the standard drug sotorasib the four predicted compounds such as ZINC001458505494, H-209, SANC00905, and PubChem CID: 11,968,893 revealed great stability and strong binding affinity for KRAS^G12C^. We hope our virtual screening protocol can be helpful to find new inhibitors against the KRAS mutants and other drug targets in the future.

### Electronic supplementary material

Below is the link to the electronic supplementary material.

Supplementary Material 1

## Data Availability

All data is available in manuscript.
