# Supplementary material for: Prospective virtual screening combined with bio-molecular simulation enabled identification of new inhibitors for the KRAS drug target
Source: BMC Chem. 2024 Mar 25;18(1):57. doi: 10.1186/s13065-024-01152-z (PMC10964497; doi:10.1186/s13065-024-01152-z)
Supplement: Supplementary file 1 — Supplementary Material 1 [file 13065_2024_1152_MOESM1_ESM.docx]

**Table S1.** Performance of ML models on the train set of independent dataset

| **Model** | **Accuracy** | **Sensitivity** | **Specificity** | **MCC** | **Precision** | **Recall** |
| --- | --- | --- | --- | --- | --- | --- |
| KNN | 0.91 | 0.98 | 0.89 | 0.80 | 0.72 | 0.84 |
| SVM | 0.92 | 0.85 | 0.94 | 0.79 | 0.88 | 0.96 |
| RF | 0.97 | 0.92 | 0.98 | 0.91 | 0.98 | 0.95 |
| GNB | 0.89 | 0.96 | 0.87 | 0.76 | 0.73 | 0.98 |

**Table S2.** Performance of ML models on the test set of independent dataset

| **Model** | **Accuracy** | **Sensitivity** | **Specificity** | **MCC** | **Precision** | **Recall** |
| --- | --- | --- | --- | --- | --- | --- |
| KNN | 0.95 | 0.97 | 0.94 | 0.88 | 0.86 | 0.92 |
| SVM | 0.89 | 0.79 | 0.93 | 0.73 | 0.81 | 0.86 |
| RF | 0.98 | 0.94 | 0.98 | 0.93 | 0.93 | 0.95 |
| GNB | 0.92 | 0.97 | 0.90 | 0.93 | 0.80 | 0.94 |


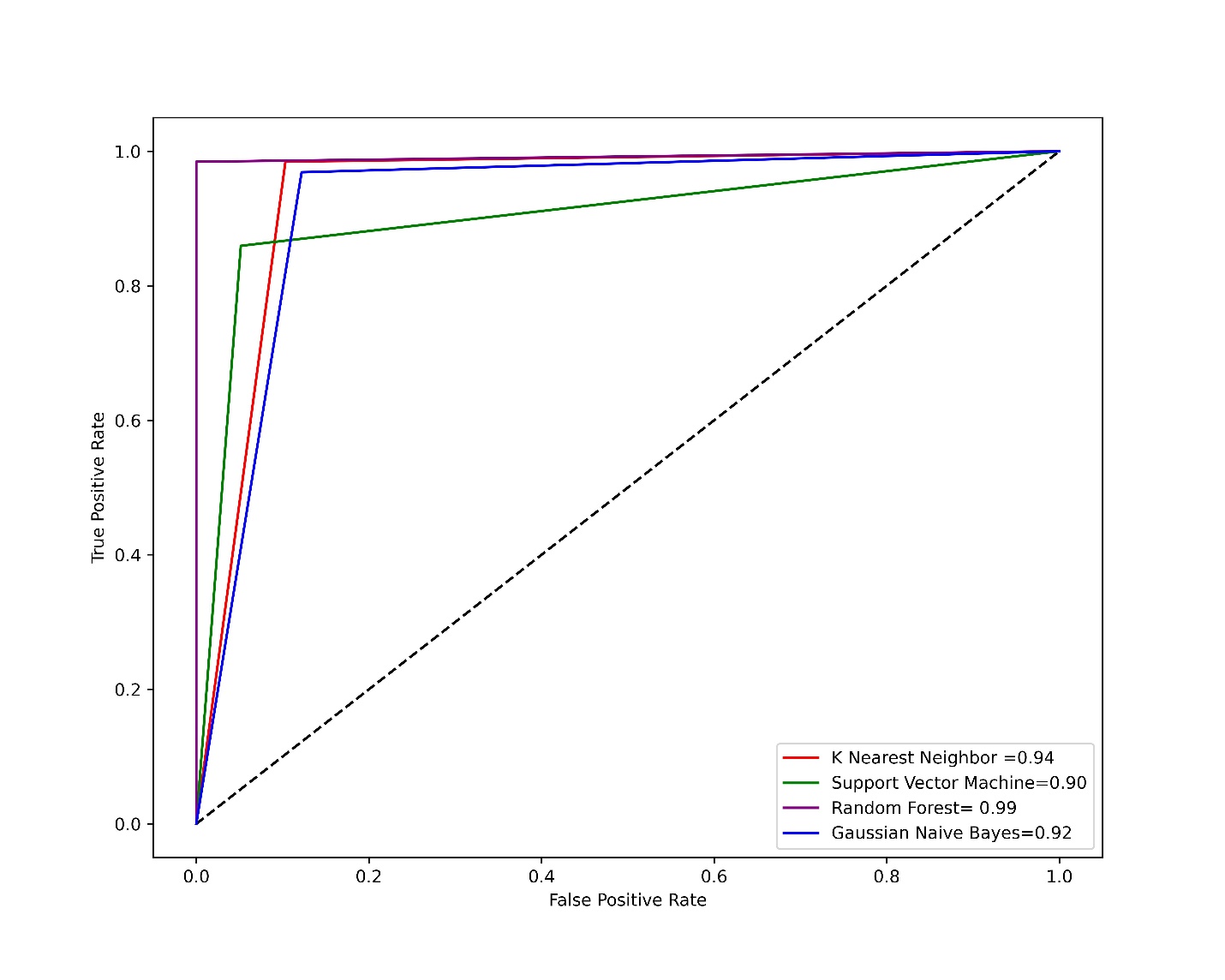


**Figure S1.** The AUC-ROC curve on the train set of independent dataset for all four models. The X-axis represents the false positive rate while the Y-axis represents the true positive rate.


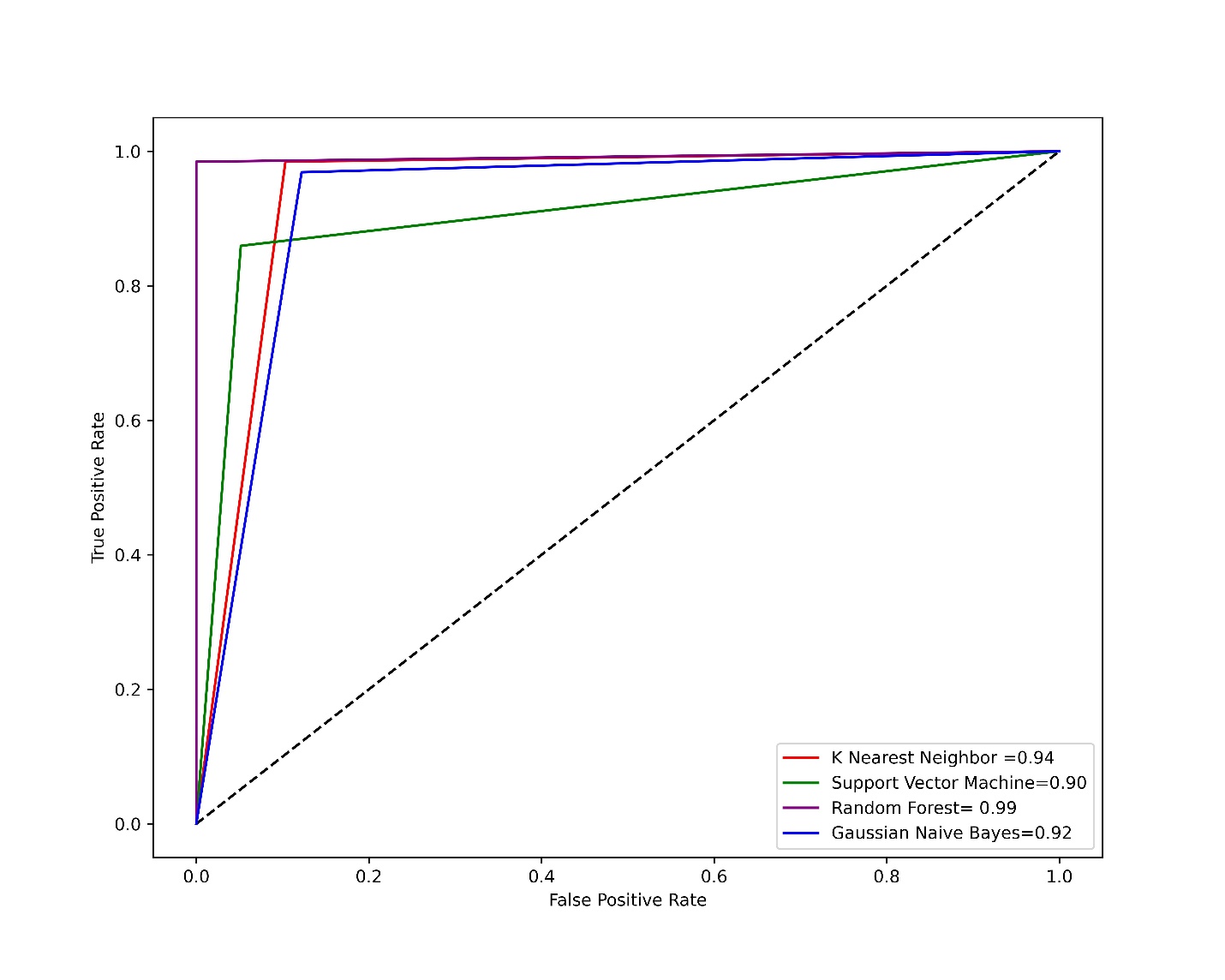


**Figure S2.** The AUC-ROC curve on the test set of independent dataset for all four models. The X-axis represents the false positive rate while the Y-axis represents the true positive rate.

**Table S3.** The docking score and interactions of the top best compounds of the ZINC database

| **Compound ID** | **Ligand** | **Receptor** | **Residues** | **Distance** | **E (kcal/mol)** | **S score** |
| --- | --- | --- | --- | --- | --- | --- |
| ZINC001436082395 | S 11  N 6  N 7  N 7  S 11 | OG  CB  CA  N  CB | SER 65  GLU 62  GLY 60  GLN 61  ALA 66 | 3.54  3.65  4.03  3.88  4.21 | -0.2  -0.6  -0.2  -0.2  -0.2 | -12.15 |
| ZINC001463157862 | N 4  C 8  C 24  C 25  O 1 | OE1  OE1  O  O  CG | GLU 62  GLN 61  GLY 13  CYS 12  LYS 88 | 3.48  3.55  3.39  3.43  3.13 | -0.5  -0.2  -0.1  -0.1  -0.1 | -11.71 |
| ZINC001463212372 | C 15  C 20  C 11  6-ring | OE2  O  5-ring  CB | GLU 62  TYR 64  HIS 95  GLU 62 | 3.35  3.54  4.45  3.54 | -0.1  -0.1  -0.2  -0.1 | -10.49 |
| ZINC001459783501 | C 16  O 19  O 19  O 19 | O  CA  N  CB | TYR 64  GLY 60  GLN 61  GLU 62 | 3.41  3.86  3.05  3.60 | -0.3  -0.4  -3.3  -0.5 | -9.20 |

**Table S4.** Drug-like properties of the top five compounds of the ZINC database

| **Compound ID** | **Toxic** | **Weight** | **logP** | **H-bond donor** | **H-bond acceptor** |
| --- | --- | --- | --- | --- | --- |
| ZINC001458505494 | NO | 450.32 | 4.63 | 1 | 3 |
| ZINC001436082395 | NO | 450.33 | 4.52 | 0 | 3 |
| ZINC001463157862 | NO | 450.40 | 3.83 | 1 | 3 |
| ZINC001463212372 | NO | 450.40 | 4.62 | 0 | 3 |
| ZINC001459783501 | NO | 450.40 | 4.89 | 0 | 3 |

**Table S5.** The docking score and interactions of the top best compounds of the in-house database

| **Compound** | **Ligand** | **Receptor** | **Residue** | **Distance** | **E (kcal/mol)** | **S score** |
| --- | --- | --- | --- | --- | --- | --- |
| H-209 | N 13  O 11  F 22  F 24  6-ring | O  ND2  NZ  NZ  CB | CYS 12  ASN 86  LYS 88  LYS 88  CYS 12 | 2.91  2.95  3.40  3.60  3.72 | -2.5  -2.1  -0.9  -0.8  -0.8 | -16.16 |
| H-237 | C 9  C 33  N 12  5-ring  6-ring | OE1  OE2  NH2  5-ring  5-ring | GLU 98  GLU 91  ARG 102  HIS 94  HIS 95 | 3.41  4.05  3.21  3.99  3.81 | -0.1  -0.1  -4.6  -0.0  -0.0 | -10.66 |
| Quinoxaline analog | C 22  CL 11  N 28  N 28 | ND1  CE  NE2  CE1 | HIS 95  LYS 88  HIS 94  HIS 95 | 3.67  3.74  3.64  3.55 | -0.1  -0.2  -0.1  -0.1 | -13.36 |
| Quinoxaline analog | C 21  N 17  N 23  N 23  N 23 | OE1  CE1  NE2  NH1  NH2 | GLU 63  HIS 95  GLN 99  ARG 102 ARG 102 | 3.41  3.33  3.59  3.91  3.20 | -0.2  -0.2  -0.1  -0.3  -1.2 | -11.30 |
| H-164 (14-Acetoxycolladonin) | C 27  C 60  O 40  O 40  O 64 | OE2  OE2  NE2  NH2  NZ | GLU 91  GLU 62  GLN 99  ARG 102  LYS 88 | 3.30  3.31  3.29  3.03  3.39 | -0.1  -0.1  -0.6  -5.5  -1.2 | -14.14 |

**Table S6.** Drug-like properties of the top four best compounds of the in-house database

| **Compound** | **Toxic** | **Weight** | **logP** | **H-bond donor** | **H-bond acceptor** |
| --- | --- | --- | --- | --- | --- |
| H-209 | NO | 363.29 | 4.63 | 1 | 3 |
| H-237 | NO | 364.43 | 4.63 | 2 | 5 |
| Quinoxaline analog | NO | 249.28 | 2.32 | 1 | 4 |
| H-164 (14 Acetoxycolladonin) | NO | 440.54 | 4.67 | 1 | 4 |

**Table S7.** The docking score and interactions of the top best compounds of the SANCDB database

| **Compound ID** | **Ligand** | **Receptor** | **Residues** | **Distance** | **Energy (Kcal/mol)** | **S Score** |
| --- | --- | --- | --- | --- | --- | --- |
| SANC00836 | O 29  O 30  O 33  6-ring | OE1  O  ND2  CB | GLU 62  CYS 12  ASN 85  CYS 12 | 3.19  2.93  3.18  4.79 | -1.8  -0.8  -0.9  -0.2 | -12.20 |
| SANC00103 | O 7  O 16  O 9  6-ring | O  OE1  ND2  CG | CYS 12  GLU 62  ASN 85  LYS 88 | 2.78  2.89  3.15  3.61 | -3.7  -0.2  -0.2  -0.5 | -9.42 |
| SANC01095 | O 17  O 15  6-ring | OE1  CG  NZ | GLU 62  LYS 88  LYS 88 | 2.92  3.56  4.43 | -6.5  -0.1  -0.2 | -8.81 |
| SANC01073 | C 6  C 8  C 17  O 20  O 19  O 20 | NE2  OE2  OE1  OE1  NH2  NH1 | HIS 95  GLU 91  GLU 63  GLU 63  ARG 102  ARG 102 | 3.55  3.51  3.57  2.88  2.96  3.12 | -0.2  -0.2  -0.2  -3.0  -3.3  -1.1 | -8.41 |
| SANC01058 | C 1  O 13 | SG  OE1 | CYS 12  GLU 62 | 3.96  2.99 | -0.3  -2.6 | -8.38 |
| SANC01068 | O 26  O 27  O 27  O 27  O 28  6-ring | OE1  OE1  CG  NZ  ND2  ND2 | GLU 62  GLU 62  LYS 88  LYS 88  ASN 86  ASN 85 | 3.45  3.12  3.65  3.29  3.05  3.93 | -0.5  -2.3  -0.1  -2.6  -0.1  -0.1 | -8.99 |

**Table S8.** Drug-like properties of the top seven best compounds of the SANCDB database

| Compound ID | Toxic | Weight | LogP | H-Bond donor | H-bond acceptor |
| --- | --- | --- | --- | --- | --- |
| SANC00905 | No | 696.79 | -1.00 | 8 | 13 |
| SANC00836 | NO | 464.38 | 0.30 | 8 | 11 |
| SANC00103 | NO | 306.27 | 0.45 | 6 | 7 |
| SANC01095 | NO | 238.19 | 1.35 | 2 | 5 |
| SANC01073 | NO | 278.44 | 6.39 | 1 | 2 |
| SANC01058 | NO | 325.98 | 2.86 | 2 | 3 |
| SANC01068 | NO | 432.38 | 0.75 | 7 | 9 |

**Table S9.** The docking score and interactions of the top best compounds of the Pakistani phytochemicals

| **PubChem CID** | **Ligand** | **Receptor** | **Residues** | **Distance** | **E (kcal/mol)** | **S score** |
| --- | --- | --- | --- | --- | --- | --- |
| 11968893 | O 5  O 12  O 10  O 10  O 11  O 12  O 14  6-ring | OE1  OE1  N  N  N  NZ  ND2  CE | GLN 61  GLU 62  GLN 61  GLU 62  CYS 12  LYS 88  ASN 86  LYS 88 | 2.77  2.56  3.99  2.81  2.87  2.85  2.83  3.67 | -3.4  -3.7  -2.8  -4.4  -4.2  -4.9  -5.4  -1.1 | -18.58 |
| 626 | O 2  O 8  O 9  N 13  O 9  N 13  C 16  C 22 | O  CG  NZ  NZ  NZ  NZ  NZ  NZ | ALA 18  LYS 16  PRO 140  LYS 165  LYS 165  LYS 165  LYS 165  LYS 165 | 2.63  3.81  3.64  2.99  2.82  2.99  2.82  3.32 | -4.5  -0.1  -0.1  -5.6  -7.8  -4.6  -5.8  -2.7 | -16.59 |
| 21637642 | C 33  C 43  O 5  O 8  O 8  O 13 | O  O  CA  CD  NZ  NZ | CYS 12  GLY 15  CYS 12  LYS 165  LYS 165  LYS 165 | 3.47  3.52  3.54  3.48  3.49  3.40 | -0.1  -0.1  -0.1  -0.4  -3.1  -0.2 | -14.59 |
| 19212 | O 7  C 18  C 24  O 4  O 6  O 6  O 4 | O  O  O  CD  CD  NZ  NH1 | GLY 138  CYS 12  CYS 12  ARG 161  LYS 165  LYS 165  ARG 161 | 2.86  3.36  3.65  3.40  3.35  2.87  3.02 | -0.4  -0.1  -0.1  -0.3  -0.1  -15.1  -4.4 | -13.72 |
| 161686 | O 17  O 12  O 15  O 15  O 19  O 11  O 19 | O  NZ  CA  NZ  NZ  NZ  NZ | TYR 137  LYS 165  ALA 18  LYS 169  LYS 169  LYS 165  LYS 169 | 2.91  3.41  3.28  3.11  2.80  3.11  2.80 | -0.5  -2.3  -0.6  -4.6  -3.0  -3.8  -6.0 | -12.30 |
| 73 | F 1  F 1  O 3  6-ring  6-ring | CE  NZ  NZ  CD  NZ | LYS 165  LYS 169  LYS 165  LYS 165  LYS 165 | 3.46  2.90  2.99  4.71  4.49 | -0.2  -2.4  -0.2  -0.3  -0.7 | -9.72 |

**Table S10.** Drug-like properties of the top best compounds of the Pakistani phytochemicals

| **Pubchem ID** | **Toxic** | **Weight** | **logP** | **H-bond donor** | **H-bond acceptor** |
| --- | --- | --- | --- | --- | --- |
| 11968893 | NO | 835.94 | 3.92 | 3 | 10 |
| 626 | NO | 394.15 | -5.88 | 1 | 7 |
| 21637642 | NO | 776.96 | 1.70 | 5 | 13 |
| 19212 | NO | 462.63 | 1.32 | 3 | 6 |
| 161686 | NO | 877.03 | 1.07 | 3 | 16 |
| 73 | NO | 401.55 | 0.99 | 1 | 3 |

**Table S11.** Docking score and interactions of covalent inhibitors with KRAS^G12C^ protein

| **Compound ID** | **H-bond interaction** | **Hydrophobic/other interaction** | **Covalent interaction** | **S Score** |
| --- | --- | --- | --- | --- |
| ZINC001458505494 | PRO 34, TYR 32  LYS 16, ALA 59 | GLN 61 | CYS 12 | -7.80 |
| SANC00905 | GLU 62, CYS 12,  GLN 99, ARG 68 | HIS 95 | CYS 12 | -9.61 |


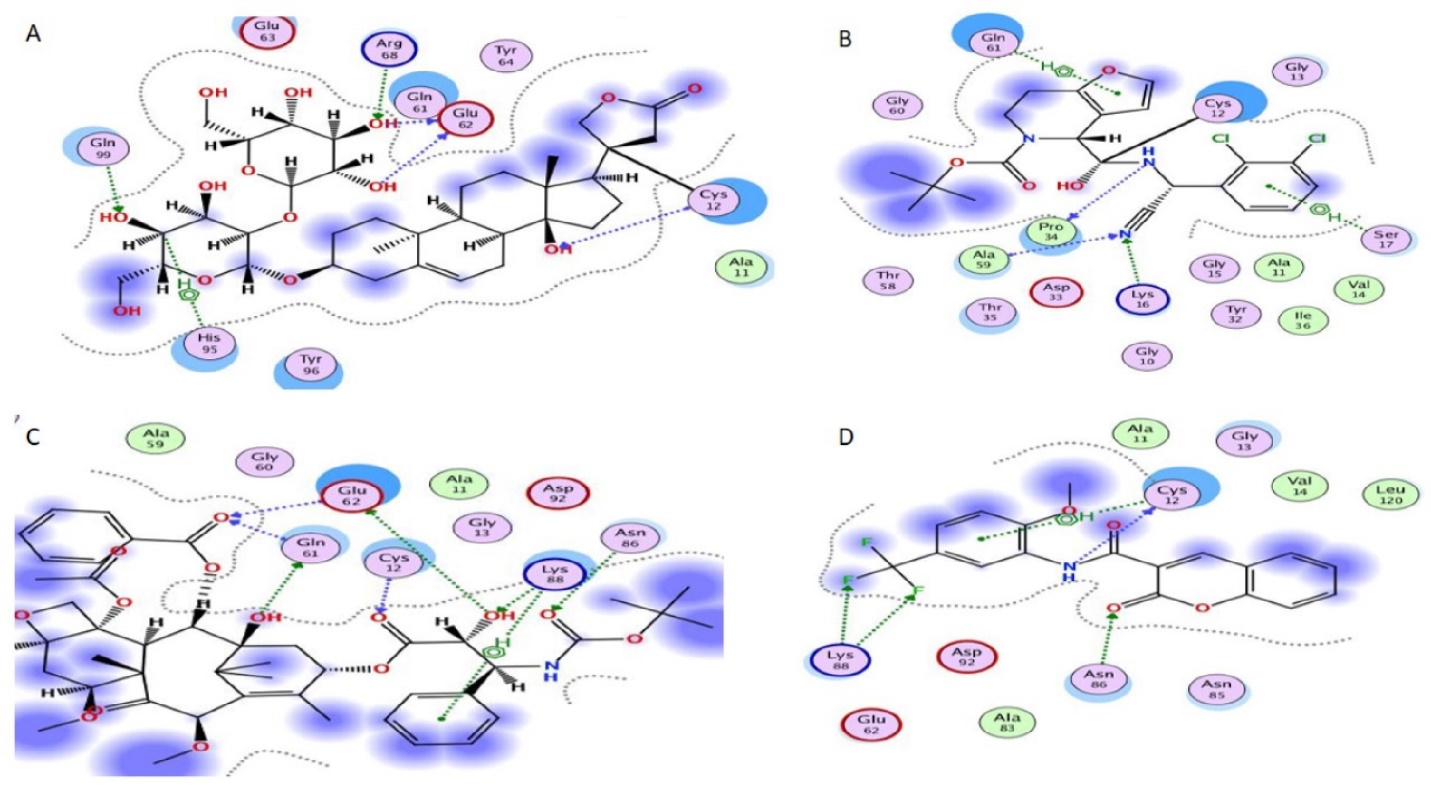


**Figure S3.** 2D interactions of (A) SANC00905 (B) ZINC001458505494 (C) PubChem CID 11968893 and (D) H-209 with the KRAS^G12C^ drug target.


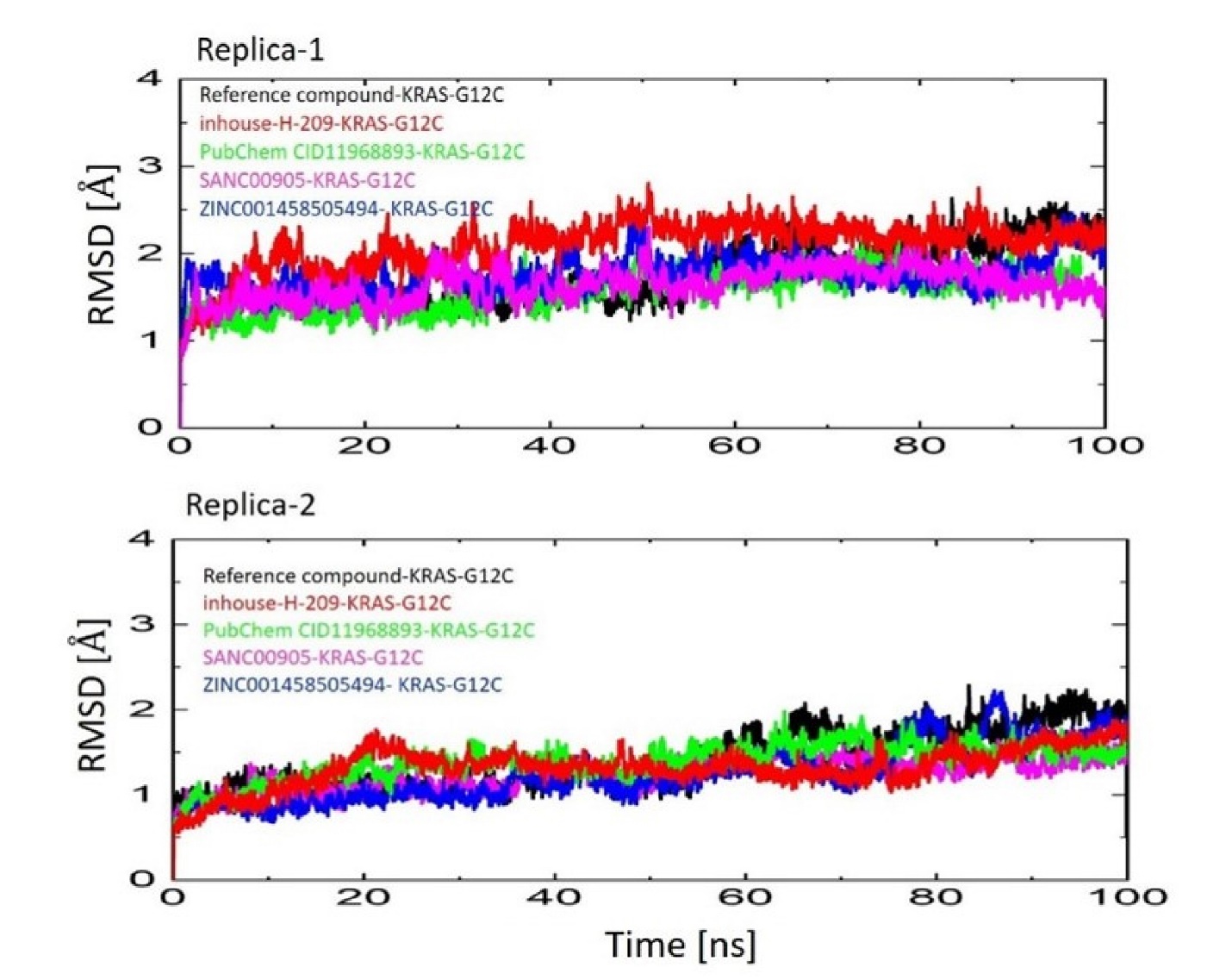


**Figure S4.** RMSD plots of Replica-1 and Replica-2 A) H-209- KRAS^G12C^ B) ZINC001458505494- KRAS^G12C^ C) PubChem-CID11968893-KRAS^G12C^ and D) SANC00905- KRAS^G12C^ in comparison with the standard drug- KRAS^G12C^ complex (Black color).


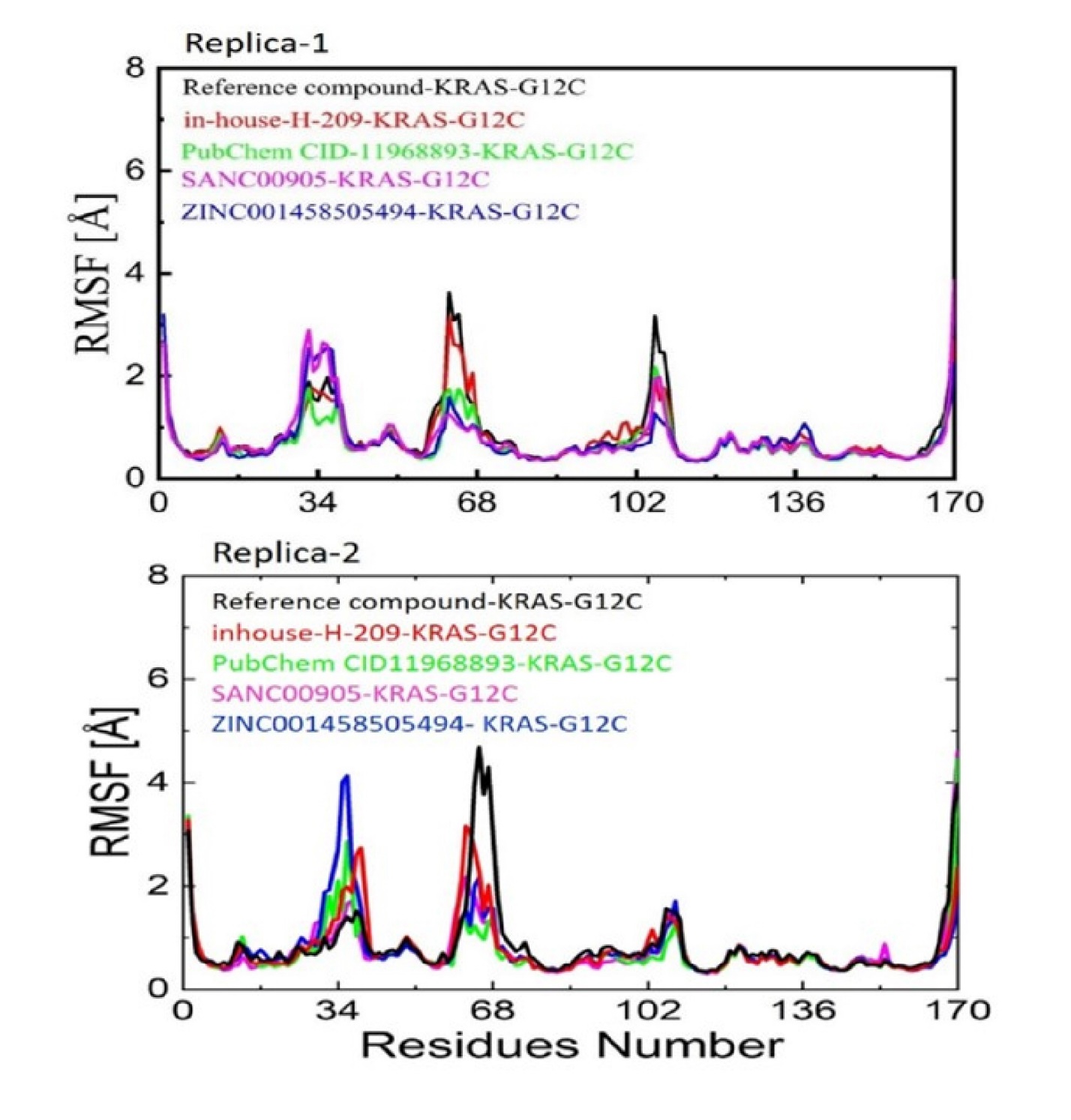


**Figure S5.** RMSF plots of the top four ligands-protein complexes and the standard drug-protein complex. H-209-KRAS^G12C^ (Red), ZINC001458505494-KRAS^G12C^ (Blue), PubChem-CID11968893-KRAS^G12C^ (green), SANC00905- KRAS^G12C^ (Magenta) and standard drug- KRAS^G12C^ complex (Black color). The number of residues is shown on the X-axis while the RMSF value is shown on the Y-axis.


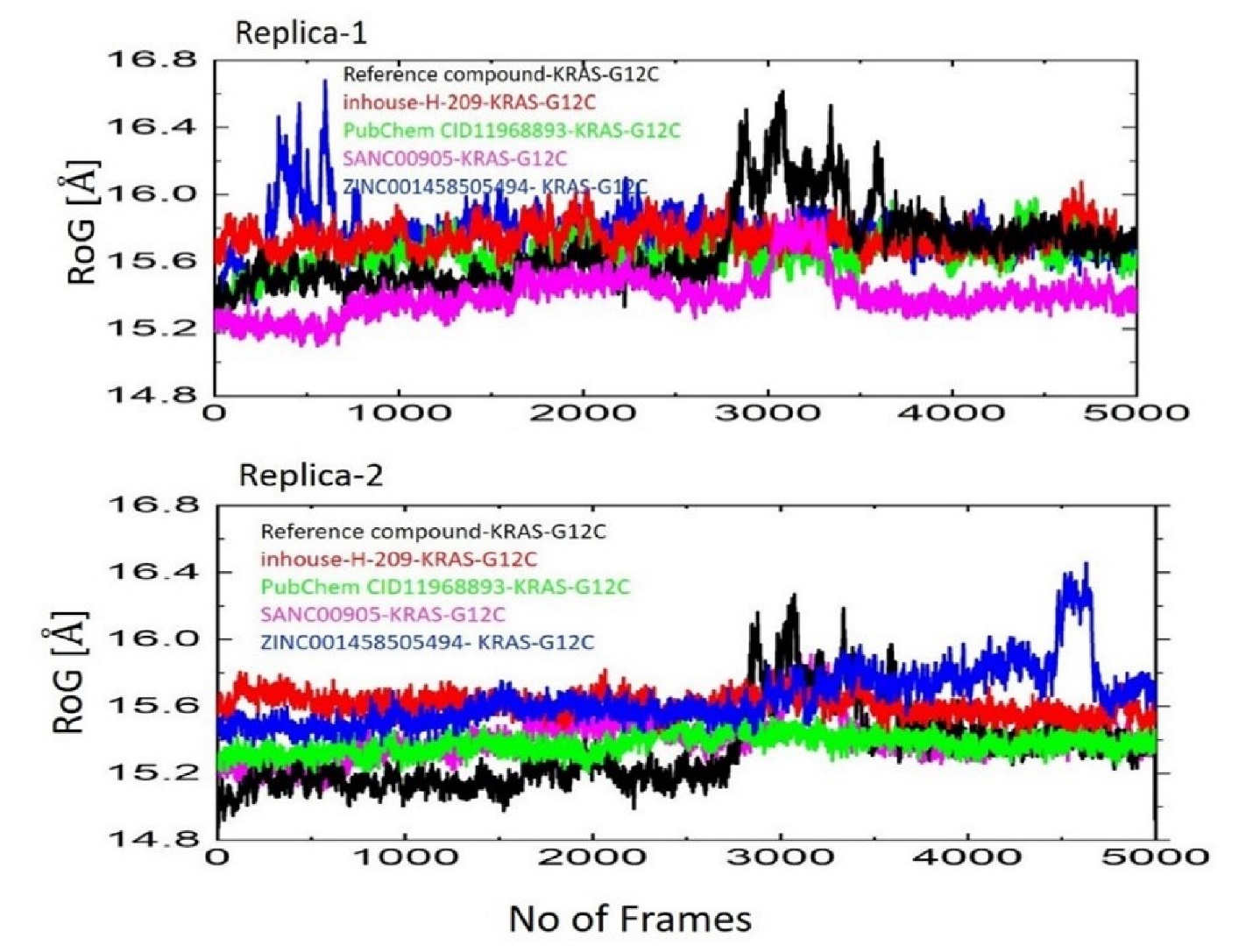


**Figure S6.** Radius of gyration (RoG) plots for Replica-1 and Replica-2 complexes A) H-209-KRAS^G12C^ B) ZINC001458505494-KRAS^G12C^ C) PubChem-CID11968893-KRAS^G12C^ and D) SANC00905- KRAS^G12C^ in comparison with the standard drug- KRAS^G12C^ complex (Black color). The X-axis displays the number of frames while the value of RoG in Angstrom is displayed on the Y-axis.
